# Intraocular pressure-lowering effects of ripasudil on open-angle glaucoma in eyes with high myopia and pathological myopia

**DOI:** 10.1038/s41598-023-49782-y

**Published:** 2023-12-21

**Authors:** Takeshi Yoshida, Sota Yoshimoto, Takuhei Nomura, Taiju Ito, Motohisa Ohno, Shintaro Yasuda, Yuto Shiotani, Kyoko Ohno-Matsui

**Affiliations:** 1 Department of Ophthalmology and Visual Science, Tokyo Medical and Dental University, 1-5-45 Yushima Bunkyo-ku, Tokyo, 1138159 Japan; 2 Department of Advanced Ophthalmic Imaging and Ophthalmology, Tokyo Medical and Dental University, 1-5-45 Yushima Bunkyo-ku, Tokyo, 1138159 Japan

**Keywords:** Diseases, Health care, Medical research, Risk factors

## Abstract

The aim is to study the intraocular pressure (IOP)-lowering effects of additional administration of ripasudil in open-angle glaucoma (OAG) patients including high myopia (HM) and pathological myopia (PM). Study design is retrospective cohort study. We assessed the changes in the mean IOP between the HM eyes (axial length ≧ 26.5 mm 33 eyes) and the non-HM eyes (axial length < 26.5 mm 29 eyes) at 4 and 12 weeks from baseline. We also assessed the IOP changes between the PM eyes (21 eyes) and the non-PM eyes (41 eyes). The significant IOP reduction by the ripasudil administration was observed at 4 weeks in the non-HM eyes and at 12 weeks in HM and non-HM eyes. And the IOP reduction in the HM eyes was significantly less than the non-HM eyes at 4 and 12 weeks. IOP reduction by ripasudil had statistically significant association with the baseline IOP and presence of PM. Furthermore, significant IOP reduction by the ripasudil administration was observed at 4 and 12 weeks in the non-PM eyes, but not in the PM eyes. The additional administration of ripasudil was effective in the HM eyes, but less than non-HM eyes. And the PM may negatively contribute to reducing the IOP by ripasudil.

## Introduction

Open angle glaucoma (OAG) is the most common type of glaucoma, characterized by a structural abnormality of the optic nerve related to retinal ganglion cell death^1,2^. Increased intraocular pressure (IOP) is known to be a main risk factor for the onset and progression of glaucoma. Therefore, lowering the IOP is the main treatment to keep visual function in patients with glaucoma^3,4^. Aqueous humor mainly drains through the conventional outflow pathway via the trabecular meshwork through the collector channel to the episcleral veins. Recently, several IOP-lowering medications, such as prostaglandin analogs, beta-blockers, carbonic anhydrase inhibitors, α2-agonists, and Rho-associated protein kinase (ROCK) inhibitors are used to lower IOP levels in glaucomatous eyes, and some of them affects the conventional outflow pathway.

ROCK inhibitors recently have been developed to reduce IOP levels in animal and human eyes^5–10^. The IOP-lowering effects of ROCK inhibitors have induced alterations in cell shape, contraction, motility, attachment, and extracellular matrix production in the trabecular meshwork and Schlemm’s canal endothelial cells, which have resulted in increase of the conventional aqueous outflow^11^. ROCK inhibitors, including ripasudil (Glanatec ophthalmic solution 0.4%; Kowa Company, Ltd., Nagoya, Aichi, Japan) is now available in the market and are used in clinical practice in a number of countries. It has been known that ripasudil increased aqueous humor outflow by expanding the juxtacanalicular trabecular meshwork^6–10^. Interestingly, some previous studies reported ripasudil has a potential to dilate episcleral veins, which may help increasing aqueous outflow^11–14^.

High myopia (HM) is characterized that the eyeball elongates excessively. The excessive elongation of the eyeball induces strong mechanical stress on all eye tissues, and this stress is associated with many severe comorbidities (such as retinal detachment, subretinal neovascularization, macular degeneration)^15^.The elongation also induces extreme thinning of the retina, choroid, and sclera membranes. In term of aqueous humor pathway, Chen et al. previously reported that HM patients have a larger Schlemm’s canal diameter and area as well as a decreased trabecular meshwork thickness using OCT^16^. However, it has not been well-understood an anatomy of the intrascleral collector channels, deep scleral plexus and episcleral veins in HM patients. Beyond the Schlemm’s canal, aqueous humor flow through the intrascleral collector channels and deep scleral plexus, and finally enter the episcleral veins^17^. Previous investigations have demonstrated marked thinning of the sclera in human myopic eyes and animal model^18–20^. The evidence indicates that the scleral thinning in HM eyes may cause lesions and obstruction in the intrascleral collector channels, deep scleral plexus, and episcleral veins, which may increase resistance distal to Schlemm’s canal and affect the effect of ripasudil treatment. However, the effects of ripasudil have not yet been well-studied in HM patents.

Among HM, pathological myopia (PM) has attracted attention in terms of various ocular complications, such as retinal detachment, retinoschisis, macular holes, posterior staphyloma, myopic choroidal neovascularization, and open-angle glaucoma (OAG)^21–24^. The PM leads to structural changes in the fundus of the eye, which can lead to loss of visual acuity. Recently an international panel of researchers in myopia uniformed classification system for PM^22,24^. In the system (the META-PM classification), myopic maculopathy lesions are newly categorized into five categories from no myopic retinal lesions (category 0), tessellated fundus only (category 1), diffuse chorioretinal atrophy (category 2), patchy chorioretinal atrophy (category 3), to macular atrophy (category 4). Lacquer cracks, myopic neovascularization, and Fuchs spot were categorized as “plus signs”. Based on the classification, PM is defined as equal to or greater than the category 2, or presence of plus lesion, or the presence of a posterior staphyloma. In eyes with PM, the deformation of eyeball is considered severe, and their retina, choroid and sclera are extremely thin, which may be structural altered; this may result in further disordered presentation of the intra-scleral collector channels, deep scleral plexus, and epi-scleral veins. Such the disorder of the conventional pathway of aqueous humor leads to IOP elevation, which may affect an effect of IOP lowering by ripasudil.

Therefore, in the present study, we evaluated the effects of ripasudil in OAG patients including HM eyes who underwent an additional dosage of ripasudil. We compared HM patients’ group with non-HM patients’ group on the effect of IOP lowering after 4 and 12 weeks from the starting of ripasudil. Furthermore, we also evaluated the association of the presence PM with the effect of IOP lowering effect by ripasudil.

## Results

Table 1 presents the patient baseline characteristics in all patients. A total of 62 patients (62 eyes; Male 25 eyes and Female 37 eyes) were analyzed in the study. Mean age was 67.5 ± 12.1 years. Mean axial length was 27.8 ± 3.0 mm. Mean CCT was 523.9 ± 35.2 µm. The eyes with HM were observed in 33 eyes. The eyes with PM were observed in 21 eyes. Mean IOP before administration of ripasudil was 15.9 ± 4.6 mmHg. Mean number of IOP-lowering medication was 3.4 ± 0.7.

**Table 1:** Total patients’ characteristics.

Preoperative data	Total patients (n = 62)
Sex (Male/Female)	25/37
Age (years)	67.5 ± 12.1
Axial length (mm)	27.8 ± 3.0
CCT (µm)	523.9 ± 35.2
HM (yes)	33
PM (yes)	21
IOP (mmHg)	15.9 ± 4.6
IOP-lowering medication number	3.4 ± 0.7

*HM* high myopia, *CCT* central corneal thickness, *IOP* intraocular pressure, PM pathological myopia, Mean ± SD.

**Figure 1:**
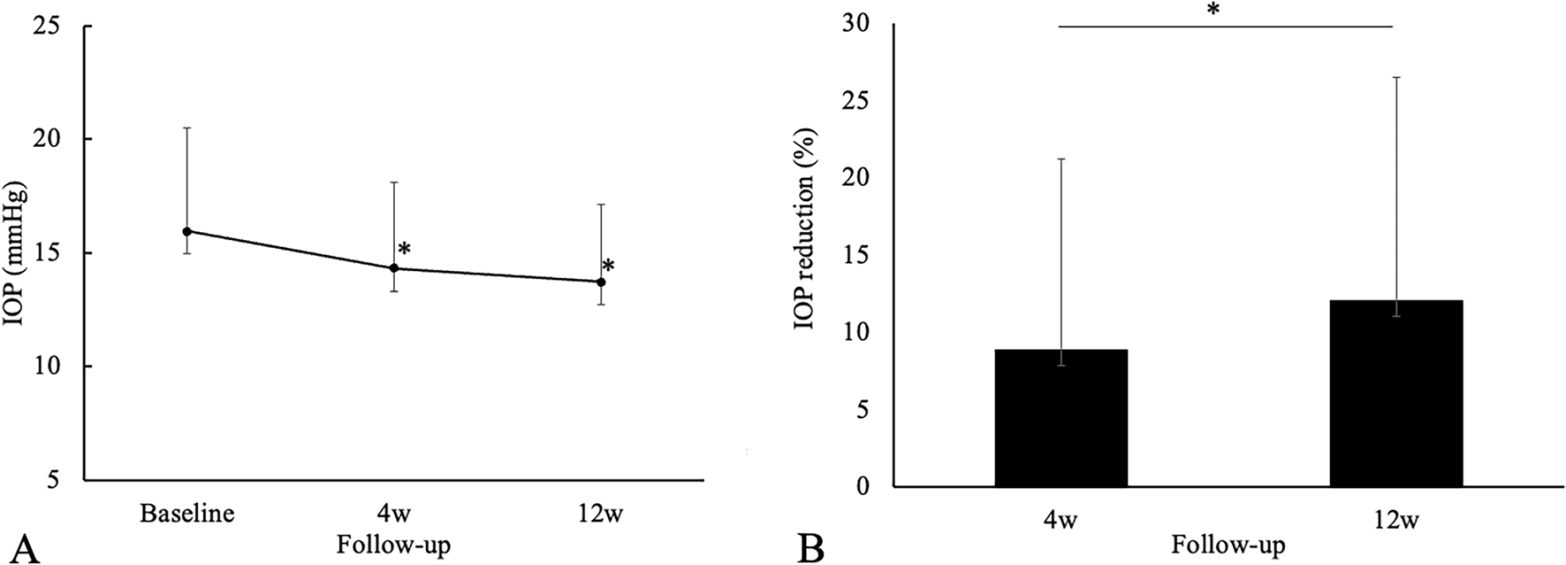
(**A**) Mean IOP ± standard deviation at 4 and 12 weeks after additional administration of ripasudil in the total eyes. A statistical comparison with the baseline was performed at each time point. (**B**) Percentage of IOP reduction from the baseline at 4 and 12 weeks after additional administration of ripasudil in the total eyes. A statistical comparison between 4 and 12 weeks was performed. *P < 0.05.

After the additional administration of ripasudil, the IOP in the total patients was decreased at 4 and 12 weeks from the baseline (Fig. 1A). The mean IOP was 15.9 ± 4.6 mmHg at baseline, while it was 14.3 ± 3.8 and 13.7 ± 3.4 at 4 and 12 weeks, respectively. There were significant differences noted for IOP values at 4 and 12 weeks compared with baseline (*P* = 0.036 and 0.003, respectively). And the average percentage of IOP reduction at 4 and 12 weeks compared to the baseline were 8.89 ± 12.39 and 12.1 ± 14.4%, respectively (Fig. 1B).

Table 2 presents the patient baseline characteristics of the HM and non-HM groups. 33 patients (33 eyes; 17 and 16 eyes in male and female, respectively) in the HM group and 29 patients (27 eyes; 8 and 21 eyes in male and female, respectively) in the non-HM group were analyzed in the study. There were no significant differences noted for gender (*P* = 0.072). Mean age was 63.2 ± 12.0 and 72.3 ± 10.6 years in the HM and non-HM groups, respectively (*P* < 0.001). Mean AXL was 30.0 ± 2.4 and 25.3 ± 1.1 mm in the HM and non-HM groups, respectively (*P* < 0.001). The presence of PM was 20 in 33 eyes in the HM group and 1 eye in the non-HM group. There were significant differences noted for the presence of PM (*P* < 0.001). Mean IOP-lowering medication number was 3.6 ± 0.5 and 3.2 ± 0.9 in the HM and non-HM groups, respectively (*P* = 0.039). There were no significant differences noted for CCT (*P* = 0.379) and IOP (*P* = 0.084) between the two groups.

**Table 2:** Patients’ characteristics in the HM and non-HM groups.

Preoperative data	HM (n = 33)	non-HM (n = 29)	*P*-value
Sex (Male/Female)	17/16	8/21	0.072*
Age (years)	63.2 ± 12.0	72.3 ± 10.6	< 0.001**
Axial length (mm)	30.0 ± 2.4	25.3 ± 1.1	< 0.001**
CCT (µm)	521.1 ± 37.7	527.1 ± 33.2	0.379**
PM (yes)	20	1	< 0.001*
Baseline IOP (mmHg)	14.9 ± 2.7	17.1 ± 6.0	0.084**
IOP-lowering medication number	3.6 ± 0.5	3.2 ± 0.9	0.039**

*HM* high myopia, *CCT* central corneal thickness, *IOP* intraocular pressure, PM pathological myopia, Mean ± SD, *P*-value: * Fisher’s exact test, **Mann–Whitney’s U test.

**Figure 2:**
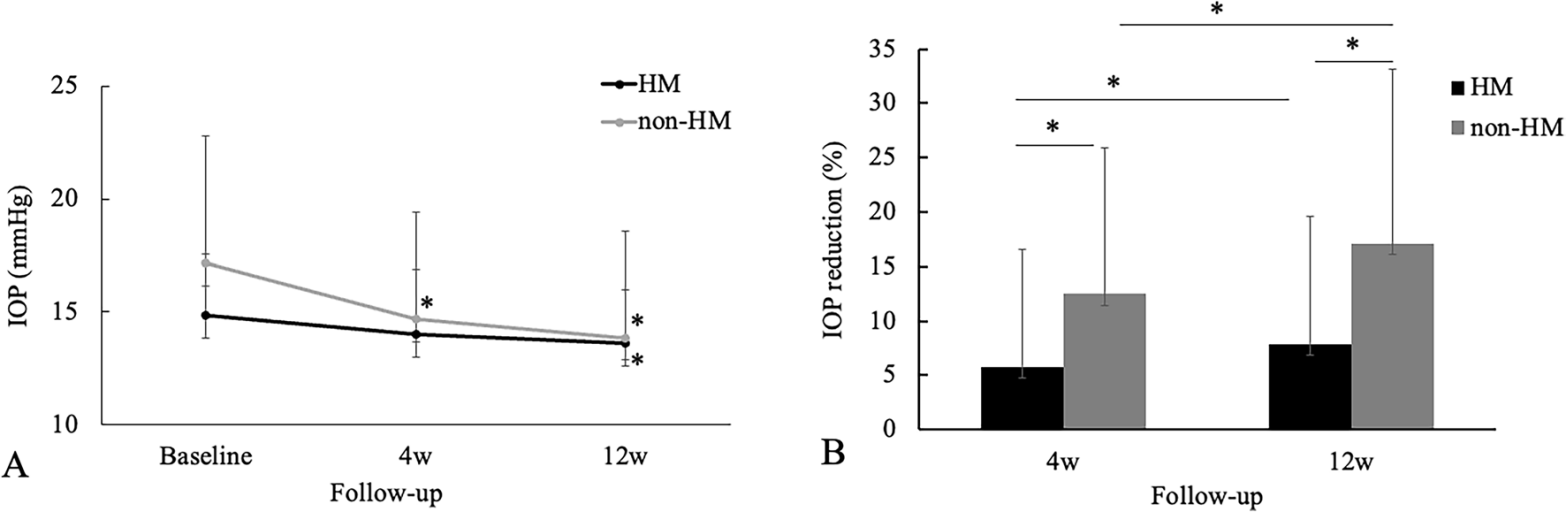
(**A**) Mean IOP ± standard deviation at 4 and 12 weeks after additional administration of ripasudil in the eyes with HM and the eyes without HM. A statistical comparison with the baseline was performed at each time point. (**B**) Percentage of IOP reduction from the baseline at 4 and 12 weeks after additional administration of ripasudil in the eyes with HM and the eyes without HM. A statistical comparison between 4 and 12 weeks was performed. *P < 0.05.

After 4 and 12 weeks from the ripasudil administration, the IOP was decreased compared in the baseline in the HM and non-HM groups (Fig. 2). The mean IOP in the HM was 14.9 ± 2.7 mmHg at baseline, while it was 14.0 ± 2.9 and 13.6 ± 2.4 at 4 and 12 weeks, respectively. In the non-HM group, the IOP was 17.1 ± 5.6 mmHg at baseline, while it was 14.7 ± 4.7 and 13.9 ± 4.7 at 4 and 12 weeks, respectively. There were significant differences noted for IOP values at 4 and 12 weeks compared with baseline in the HM group (*P* = 0.005 and < 0.001, respectively). And There was significant difference noted for IOP values at 12 weeks compared with baseline in the non-HM group (*P* < 0.001). However, there was no significant difference noted for IOP values at 4 weeks compared with baseline in the non-HM group (*P* = 0.082). In addition, there were no significant differences noted for IOP values at 4 and 12 weeks between the HM and non-HM group (*P* = 0.852 and 0.706, respectively.). And the average percentage of IOP reduction at 4 and 12 weeks compared to the baseline in non-HM group were 12.47 ± 13.42 and 17.11 ± 16.01%, respectively. The average percentage of IOP reduction at 4 and 12 weeks compared to the baseline in HM group were 5.70 ± 10.84 and 7.86 ± 11.76%, respectively. There were significant differences between the non-HM and HM groups in the percentage reduction of IOP by ripasudil at 4 and 12 weeks (*P* = 0.042 and 0.023, respectively)

**Figure 3:**
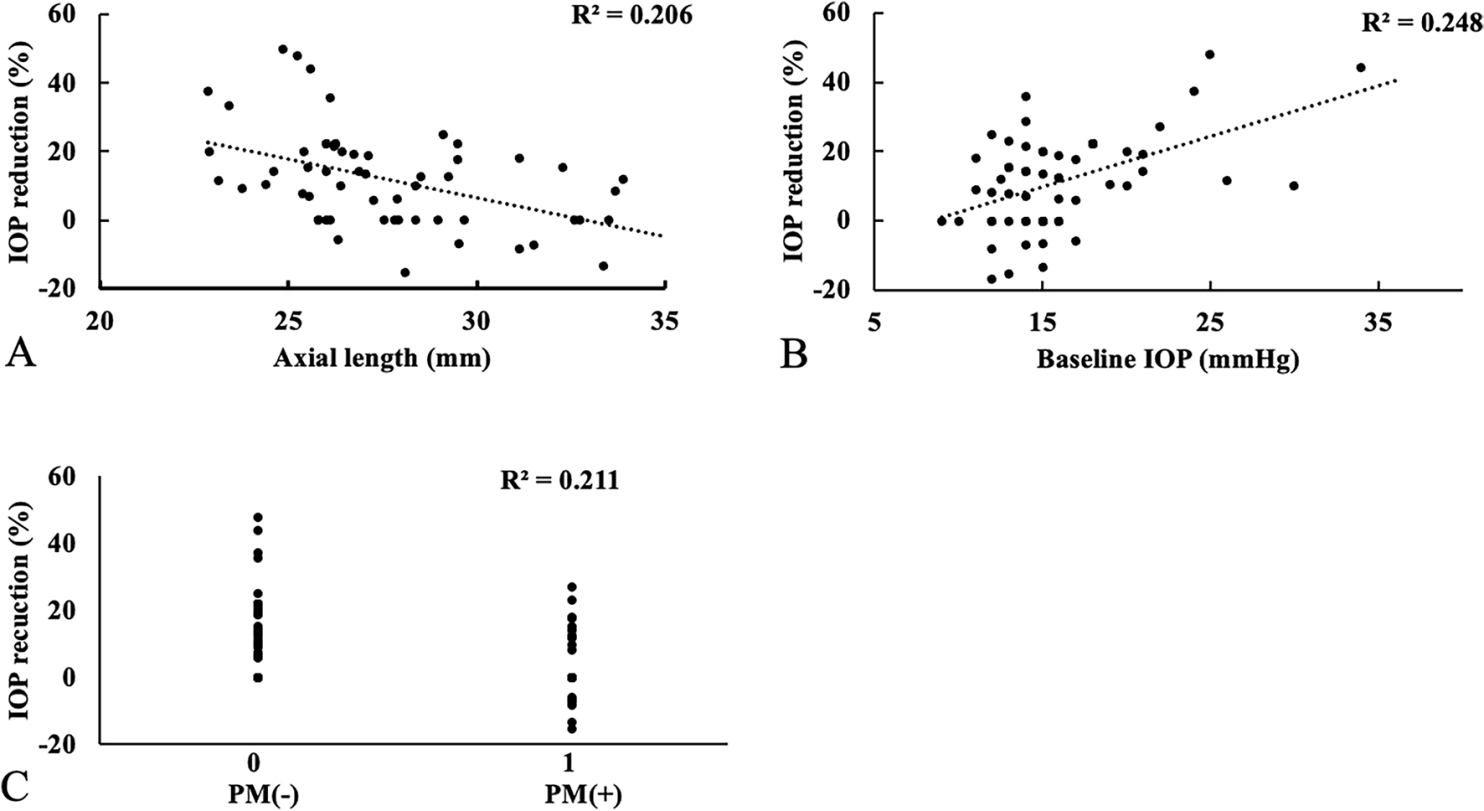
Scatterplots showing the relationship between IOP reduction by ripasudil and axial length (**A**), baseline IOP (**B**), and presence of PM (**C**). *R*^2^ is the coefficient of determination.

The results of the linear regression analyses for all patients are illustrated in Table 3. In the univariate analysis, the AXL, baseline IOP and presence of PM were statistically associated with IOP reduction at 12 weeks by the additional administration of ripasudil. The relationship between the percentage of IOP reduction at 12 months and the AXL was shown in Fig. 3A (global model fit R^2^ = 0.206). The relationship between the percentage of IOP reduction at 12 months and the baseline IOP was shown in Fig. 3B (global model fit R^2^ = 0.248). Figure 3C demonstrates the relationship between the percentage of IOP reduction at 12 months and the presence of PM (global model fit R^2^ = 0.211). In the multivariate analysis, there were statistically significant association with the IOP reduction in the baseline IOP and presence of PM (95% CI 0.336–1.810, *p* = 0.005 in baseline IOP and 95% CI − 21.79 to − 1.297, *p* = 0.028 in the presence of PM).

**Table 3:** Results of univariate and multivariate risk factor analysis for percentage of IOP reduction by ripasudil at 12 weeks.

	Univariate		Multivariate	
	*P* value	95% CI	*P* value	95% CI
Sex (Male/Female)	0.690	− 9.109 to 6.069		
Age (years)	0.889	− 0.330 to 0.287		
Axial length (mm)	0.029	− 2.502 to − 0.138	0.528	− 1.134 to 2.187
CCT (µm)	0.492	− 0.142 to 0.069		
IOP-lowering medication number	0.132	− 9.166 to 1.228		
Baseline IOP (mmHg)	< 0.001	0.562–2.041	0.005	0.336–1.810
PM	0.001	− 18.640 to − 4.691	0.028	− 21.790 to − 1.297

*CI* confidence interval, *HM* high myopia, *CCT* central corneal thickness, *IOP* intraocular pressure, *PM* pathological myopia, Mean ± SD.

Table 4 presents the patient baseline characteristics of the individuals with PM and the ones without PM. 21 patients (21 eyes; 10 and 11 eyes in male and female, respectively) in the individuals with PM and 41 patients (41 eyes; 15 and 26 eyes in male and female, respectively) in the ones without PM were analyzed. There were no significant differences noted for sex (*P* = 0.4528). Mean AXL was 31.1 ± 2.4 and 26.1 ± 1.7 mm in the individuals with PM and the ones without PM, respectively (*P* < 0.001). Mean baseline IOP was 14.1 ± 2.5 and 16.9 ± 5.2 in the individuals with PM and ones without PM, respectively (*P* = 0.017). There were no significant differences noted for Age (*P* = 0.4396), CCT (*P* = 0.097), and number of IOP-lowering medication (*P* = 0.4400) between the two groups.

**Table 4:** Patients’ characteristics in the individuals with PM and ones without PM.

Preoperative data	PM(n = 21)	non-PM (n = 41)	*P*-value
Sex (Male/Female)	10/11	15/26	0.453*
Age (years)	66.8 ± 11.5	67.8 ± 12.7	0.440**
Axial length (mm)	31.1 ± 2.4	26.1 ± 1.7	< 0.001**
CCT (µm)	515.8 ± 28.1	528.1 ± 38.4	0.097**
Baseline IOP (mmHg)	14.1 ± 2.5	16.9 ± 5.2	0.017**
IOP-lowering medication number	3.5 ± 0.5	3.3 ± 0.8	0.440**

*HM* high myopia, *CCT* central corneal thickness, *IOP* intraocular pressure, PM pathological myopia, Mean ± SD, *P*-value: * Fisher’s exact test, **Mann–Whitney’s U test.

**Figure 4:**
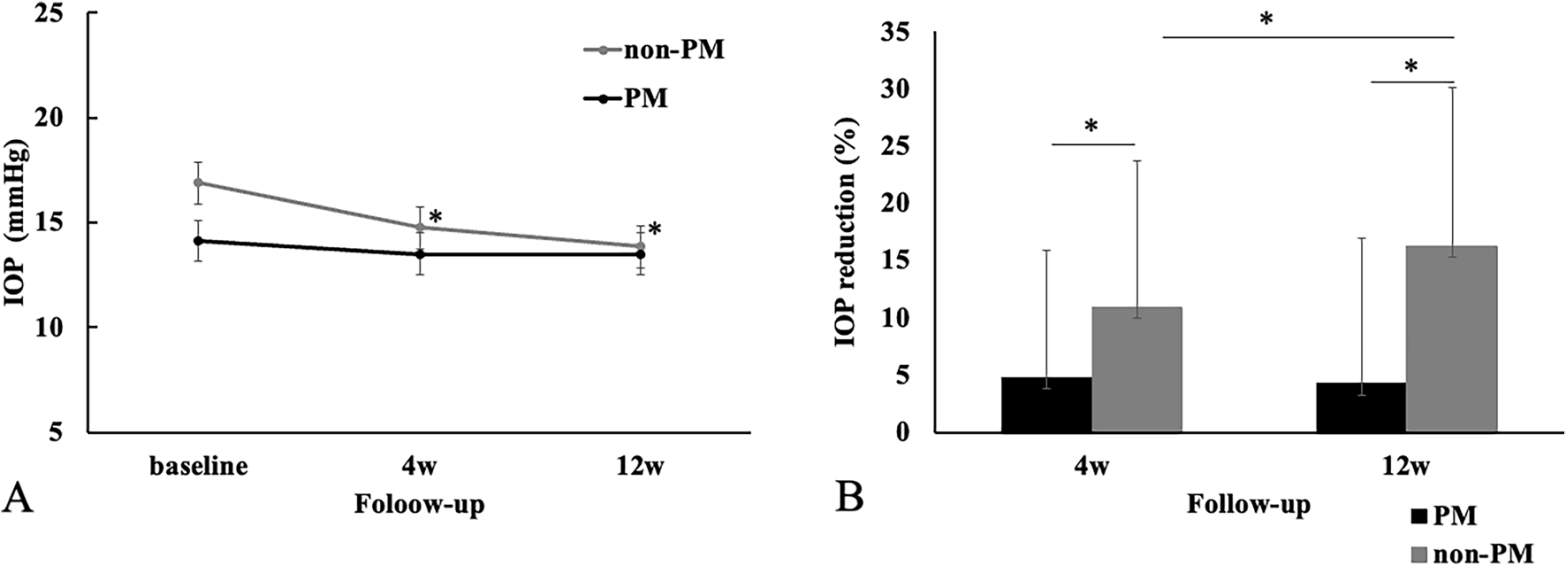
(**A**) Mean IOP ± standard deviation at 4 and 12 weeks after additional administration of ripasudil in the eyes with PM and the eyes without PM. A statistical comparison with the baseline was performed at each time point. (**B**) Percentage of IOP reduction from the baseline at 4 and 12 weeks after additional administration of ripasudil in the eyes with PM and the eyes without PM. A statistical comparison between 4 and 12 weeks was performed. *P < 0.05.

Figure 4 shows the IOP-lowering effect by the additional administration of ripasudil in the eyes with PM and the ones without PM at the at 4 and 12 weeks from the baseline. The mean IOP in the individuals with PM was 14.1 ± 2.5 mmHg at baseline, while it was 13.5 ± 3.1 and 13.5 ± 2.7 at 4 and 12 weeks, respectively (Fig. 4A). There were no significant differences noted for IOP values at 4 and 12 weeks compared with baseline (*P* = 0.468 and 0.427, respectively). In the individuals without PM, the IOP was 16.9 ± 5.2 mmHg at baseline, while it was 14.8 ± 4.1 and 13.9 ± 3.8 at 4 and 12 weeks, respectively. There were significant differences noted for IOP values at 4 and 12 weeks compared with the one of baseline (*P* = 0.044 and 0.004, respectively). In addition, there were significant differences noted for IOP values between the two groups at baseline (*P* = 0.017), and there were no significant differences noted for IOP values between the two groups at 4 and 12 weeks between the two groups (*P* = 0.166 and 0.979, respectively.) The average percentage of IOP reduction at 4 and 12 weeks compared to the baseline in eyes with non-PM were 10.96 ± 12.73 and 16.27 ± 13.81%, respectively (Fig. 4B). The average percentage of IOP reduction at 4 and 12 weeks compared to the baseline in eyes with PM were 4.78 ± 11.16 and 4.22 ± 12.79%, respectively. There were significant differences between the non-HM and HM groups in the percentage reduction of IOP by ripasudil at 4 and 12 weeks (*P* = 0.024 and 0.012, respectively).

## Discussion

Our results demonstrated that IOP reduction by additional administration of ripasudil had significant IOP-lowering effect in the eyes with HM and non-HM, however; the IOP reduction rate in the eyes with HM was smaller than the non-HM. And a good IOP reduction by ripasudil was associated with the higher baseline IOP. However, the IOP reduction by ripasudil was not effective in the eyes with PM. To the best of our knowledge, this is the first study to examine IOP reduction by ripasudil in OAG patients with HM and PM.

The IOP-lowering efficacy of ripasudil has been studied in eyes with POAG or ocular hypertension (OHT). Tanihara et al. conducted a phase 2 ripasudil clinical trial in 210 patients with POAG or OHT. The trial found that ripasudil lowered mean IOP by 3.5 mmHg at before instillation (trough) and by 4.5 mmHg after 2 h from instillation (peak)^25^. In a report from the Clinical Study Group in Japan, they performed a prospective study to investigate the IOP-lowering effects of ripasudil in 173 patients with POAG, OHT, or pseudoexfoliative (PE) glaucoma, showed that ripasudil monotherapy reduced mean IOP from baseline by 2.6 mmHg at trough and 3.7 mmHg at peak^25^. The effects of ripasudil in combination with these drugs were also tested. The results from a pair of phase 3 trials (the Ripasudil–Timolol Study and Ripasudil–Latanoprost Study) comprising 413 patients with POAG or OHT showed that the mean reduction in IOP from baseline was 2.4 mmHg at trough and 2.9 mmHg at peak after the addition of ripasudil to timolol, and 2.2 mmHg at trough and 3.2 mmHg at peak after the addition of ripasudil to latanoprost^26^. Inoue et al. prospectively assessed the effect of the addition of ripasudil to latanoprost on IOP in 26 patients with POAG or normal tension glaucoma (NTG), mean IOP significantly changed from 16.7 mmHg at baseline to 15.5 mmHg at 1 month and 14.9 mmHg at 3 months^27^. And Tanihara et al. demonstrated that eyes with NTG showed a statistically significant reduction in IOP (11.0%, from 14.6 ± 3.2 to 13.0 ± 2.9 mm Hg) with ripasudil at 3 months^28,29^. Sakata et al. found that eyes with NTG had a statistically significant reduction in IOP (13.3%, from 13.07 ± 1.21 mmHg) with ripasudil at 3 months^30^. Maruyama et al. demonstrated a statistically significant reduction in IOP in eyes with NTG at 3 months (-14.2%, from 15.1 ± 4.6 to 13.3 ± 3.7 mmHg)^31^. There is still limited previous research on the intraocular pressure-lowering effects of Ripasudil with NTG. However, these studies have demonstrated significant intraocular pressure reduction even with NTG. The efficacy of additional use of ripasudil has been examined in other clinical studies. Inoue et al. retrospectively analyzed 119 eyes with POAG, OHT, or NTG treated with an average of 3.8 anti-glaucoma medications and reported that the addition of ripasudil significantly lowered mean IOP from 19.8 mmHg at baseline to 17.5 mmHg at 1 month and 16.8 mmHg at 3 months^32^. Thus, many studies demonstrated IOP-lowering effect by ripasudil tended to become maximum after several months from the beginning of administration. In the present study, we demonstrated the mean IOP in total patients with POAG was significantly decreased to 14.3 ± 3.8 and 13.7 ± 3.4 at 4 and 12 weeks from the baseline (15.9 ± 4.6 mmHg) by additional administration of ripasudil, and the rate of IOP reduction were 8.9 and 12.1%, respectively, which looks less IOP-lowering effect by ripasudil than the ones in previous studies^26,27,32–35^. However, the average percentage of IOP reduction at 4 and 12 weeks compared to the baseline in non-HM group were 12.5 and 17.1%, respectively, which has a good agreement with the previous studies^26,27,32–35^. On the other hand, the average percentage of IOP reduction at 4 and 12 weeks compared to the baseline in HM group were 5.7 and 7.9%, respectively. And there were significant differences between the non-HM and HM groups in the reduction rate of IOP from the baseline by ripasudil at 4 and 12 weeks. We should pay attention to the eyes with HM when we administrate ripasudil, however; the additional administration of ripasudil to the eyes with HM is still effective on IOP-lowering and promising further IOP reduction. And the IOP-lowering effect by ripasudil at 12 weeks was more effective more than ones at 4 weeks in HM and non-HM eyes, as the previous studies demonstrated^26,27,32^, which indicates it needs to keep monitoring of IOP-lowering effect at least 12 weeks.

The one of research characteristics in the present study is that a certain number of eyes with HM were included. HM has recently attracted attention in terms of high incidence of various ocular complications including OAG^21–24^. Recently, Wang et al. reported that HM was a major risk factor for the development of OAG with a 7.3-fold risk increase as compared with emmetropic eyes in a 10-year follow-up^36^. And we also previously demonstrated that significant VF defects were newly developed in 13.2% of HM eyes during a mean follow-up of 11.6 years^37^. Myopia is growing around the world, with a recent study estimating that on average, 30% of the world is currently myopic and by 2050, almost 50% will be myopic, that’s a staggering 5 billion people^38^, which indicates HM also may be growing and OAG patients may be increasing. Hence, it is important to understand the efficacy of IOP-lowering drug including ripasudil in HM eyes. Furthermore, in the eyes with HM, PM often occurs has been recently attracted highly attention in terms of PM-associated ocular complications^21,22,39,40^. PM differs distinctly from HM regarding a range of parameters. HM is characterized by a high degree of myopic refractive error, whereas PM is defined by the presence of typical complications in the posterior fundus. Refractive error or axial length alone often does not adequately reflect PM^41^. Indeed, the multivariate analysis in the present study demonstrated the presence of PM was significantly associated with the efficacy of ripasudil, but not with AXL, which suggests some structural change may be associated with the IOP-lowering effect by ripasudil. In the current META-PM classification, PM is defined according to a categorization that is equal to or greater than diffuse myopic maculopathy, plus posterior staphyloma formation^22^, and it is possible that the greater grade of maculopathy and the presence of posterior staphyloma may have the greater structural changes in retina, choroid and sclera due to the elongation and deformation of eyeball. The greater structural changes in PM often induce serious and irreversible PM-associated ocular complications^21,22,39,40^. And we recently reported that IOP-lowering effect by trabeculotomy using Kahook Dual Blade in the HM eyes with PM was lesser than the HM eyes without PM, and we speculated the less efficacy may result from abnormality of aqueous humor outflow in sclera of the eyes with PM^42^. In the present study, we also demonstrated that the presence of HM, especially PM, was a risk of less IOP-lowering effect by ripasudil. We speculate that poor outcomes may have several causes. The greater grade of maculopathy and the presence of posterior staphyloma in the eyes with PM may have the greater structural changes in gonio and sclera as well as retina and choroid. Chen et al. recently demonstrated the eyeball elongation was accompanied by an enlarged Schlemm’s canal diameter and decreased trabecular meshwork using OCT^16^. They also suggested that the thinner trabecular meshwork may result in less resistance of aqueous out flow at the site of trabecular meshwork. The structural changes in trabecular meshwork in the eyes with HM, especially PM, may result in poor IOP-lowering effect by ripasudil, because the aqueous outflow resistance may be already low prior to ripasudil administration that decreases resistance of aqueous humor out flow by expanding the juxtacanalicular trabecular meshwork^6–10^. Another possibility is abnormalities in the main aqueous outflow beyond the trabecular meshwork in the eyes with HM and PM. Aqueous humor goes through the intrascleral collector channels and the deep scleral plexus course through the sclera to the episcleral veins^17^, and deformed and irregular eye shapes in HM and PM may cause lesions and obstruction in the main aqueous outflow beyond the trabecular meshwork. Especially in HM eyes with PM, the deformation of eyeball is considered severe, and their sclera may be structural altered. Previous investigations have demonstrated marked thinning of the sclera, choroid, and retina in HM with stretching of the sclera, choroid, and retina caused by axial length elongation^18–20^; this may result in a disordered presentation of the intra-scleral collector channels, deep scleral plexus, and epi-scleral veins. Ripasudil has a potential to dilate episcleral veins, which may help increasing aqueous outflow and decreasing IOP^11–14^. In addition, we recently analyzed the trabeculotomy surgical outcome in eyes with HM and PM, and the IOP-lowering in the eyes with HM and PM was less effective than ones in the non-HM eyes^42^, which may result from the structural changes in sclera due to HM and PM. These structural changes may contribute to the less IOP-lowering effect by ripasudil seen in the eyes with HM and PM. Presumably, these findings require additional investigation. Relevant histological studies would help answer these questions more comprehensively. And ripasudil should be included in a list of additional IOP-lowering medicine in the eyes with HM, because the significant IOP lowering by ripasudil was demonstrated in the present study. And the presence of PM may be the most important factors predicting IOP-lowering effect by ripasudil in the eyes with OAG.

There were several limitations to this study. First, our study is limited by its relatively small sample size, which might restrict the power to identify significant associations, such as with the IOP-lowering effect by ripasudil and presence of PM. No study has evaluated the correlation between pre-administration clinical parameters (such as baseline IOP, AXL, HM and PM) and IOP-lowering effect by ripasudil ever, and our study is the first investigation about them. Further investigation with larger samples is necessary for complete understanding in the association between the parameters and IOP-lowering effect by ripasudil. Second, our study is limited by its relatively short-term investigation. Glaucoma patients need to continuously administrate IOP-lowering drugs to prevent from visual field loss for long time. The previous study demonstrated the PM become advanced with aging^15^, which indicates the structure of aqueous humor outflow pathway may be influenced by aging. Longer-term study is also needed for complete understanding the IOP-lowering effect by ripasudil. Third, we did not account for the variability in drug adherence. Previous study showed approximately half of all glaucoma patients discontinued glaucoma medications within 6 months^43^. But this bias would be small, because we included only patients who visited ophthalmologists on schedule for 12 weeks, who are more inclined to comply with treatment than the general population. Fourth, distinguishing myopic changes from glaucoma is a significant challenge. Consequently, participants exhibiting glaucomatous visual field abnormalities were classified as OAG patients in the current study. However, at the very least, we excluded the participants with VF disorder due to retinal disorder associated with HM such as chorioretinal atrophy, retinal schisis, and myopic CNV, thereby minimizing potential issue.

In conclusion, our results suggest that in OAG patients with HM, the additional administration of ripasudil significantly reduced IOP, but less than the ones with non-HM eyes. And the presence of PM negatively contributed to reducing the IOP. Our result may become important in a decision of applying ripasudil treatment in HM and PM patients. Especially, we should pay more attention the presence of PM before administrating ripasudil to OAG patients.

## Methods

### Ethics statement

This study was approved by the ethics committee of Tokyo Medical and Dental University (study approval no.: M2020-161) and adhered to the tenets of the Declaration of Helsinki. A retrospective review of medical records was approved by the institutional review board of Tokyo Medical and Dental University. All subjects gave their written informed consent to participate to the study following an explanation of the nature of the study.

### Study design and patients

This analysis was a retrospective, single-center, observational, comparative clinical study performed at the high myopia and glaucoma outpatient center in Tokyo Medical and Dental University (TMDU) Hospital between 2017 and 2020. Patients were examined at the high myopia and glaucoma center of TMDU hospital and considered for admission into the study if they had OAG diagnosis and additional dosage of ripasudil. We reviewed the medical charts of 62 consecutive OAG patients who had additional administration of ripasudil. Patients were followed up for at least 12 weeks after the starting of ripasudil treatment. We obtained information on medical history, Goldmann applanation IOP (mmHg), axial length (AXL; mm), Central cornea thickness (CCT; µm), and number of glaucoma medications, from patients’ medical records. PM discrimination was performed using the PM classification system, as described previously^24^. Exclusion criteria were as follows: patients with history of intraocular surgery except cataract surgery, a history of incisional glaucoma surgery, use of additional anti-glaucoma agents except ripasudil during a post dosage 12-week period and clinically other typed glaucoma. And the eyes with primary angle closure glaucoma and secondary glaucoma, such as exfoliation glaucoma, steroid-induced glaucoma, neovascular glaucoma, were not included in the present study. In case that ripasudil was administrated in both eyes, one eye was randomly selected and enrolled in this study.

The primary outcome measure was the mean IOP change. We analyzed the change in the IOP values and percentage of IOP reduction by ripasudil at 4 and 12 weeks from the starting of ripasudil. Furthermore, we divided the patients into HM group (AXL; equal to and more than 26.5 mm) and non-HM group (AXL; less than 26.5 mm), and we analyzed in the same manner. We also divided the patients into PM group (eyes with PM) and non-PM group (eyes without PM), and we analyzed in the same manner. We assessed the relationship between the change in IOP by ripasudil and the ocular background factors in all patients.

### Statistical analysis

All statistical analyses were performed using PRISM for Mac (GraphPad Software., Boston, MA, USA). Comparisons of clinical characteristics between the HM and non-HM groups were performed using the Mann–Whitney U test for continuous variables and Fisher’s exact test for categorical variables. Changes from baseline were compared using paired samples t-test. The following factors were tested for associations with the decrease of IOP: sex, age, baseline IOP, CCT, AXL, number of IOP-lowering medication, and presence of PM. Univariate and multivariate linear regression analyses were performed to identify variables associated with value of IOP-lowering by the administration of ripasudil at 12-week from the baseline. If f* P* < 0.05, the factors were used in multivariate analysis. A *P*-value of < 0.05 was considered the threshold for statistical significance. All statistical values are presented as means ± standard deviations (SD).

## Data Availability

The datasets used and analyzed during the current study available from the corresponding author on reasonable request.

## References

[1] Quigley HA (2011). Glaucoma. Lancet.

[2] Weinreb RN, Aung T, Medeiros FA (2014). The pathophysiology and treatment of glaucoma: A review. JAMA.

[3] Cohen LP, Pasquale LR (2014). Clinical characteristics and current treatment of glaucoma. Cold Spring Harb. Perspect. Med..

[4] Mantravadi AV, Vadhar N (2015). Glaucoma. Prim. Care.

[5] Bacharach J (2015). Double-masked, randomized, dose-response study of AR-13324 versus latanoprost in patients with elevated intraocular pressure. Ophthalmology.

[6] Tanihara H (2013). Phase 2 randomized clinical study of a Rho kinase inhibitor, K-115, in primary open-angle glaucoma and ocular hypertension. Am. J. Ophthalmol..

[7] Tanihara H (2015). Intra-ocular pressure-lowering effects of a Rho kinase inhibitor, ripasudil (K-115), over 24 h in primary open-angle glaucoma and ocular hypertension: A randomized, open-label, crossover study. Acta Ophthalmol..

[8] Honjo M, Tanihara H (2018). Impact of the clinical use of ROCK inhibitor on the pathogenesis and treatment of glaucoma. Jpn. J. Ophthalmol..

[9] Tanihara H (2019). Safety and efficacy of ripasudil in japanese patients with glaucoma or ocular hypertension: 3-month Interim analysis of ROCK-J, a post-marketing surveillance study. Adv. Ther..

[10] Kaneko Y (2016). Effects of K-115 (ripasudil), a novel ROCK inhibitor, on trabecular meshwork and Schlemm’s canal endothelial cells. Sci. Rep..

[11] Sato T, Kawaji T (2021). Effects of ripasudil on open-angle glaucoma after circumferential suture trabeculotomy ab interno. J. Clin. Med..

[12] Akagi T (2020). Short-term effects of different types of anti-glaucoma eyedrop on the sclero-conjunctival vasculature assessed using anterior segment OCTA in normal human eyes: A pilot study. J. Clin. Med..

[13] Kiel JW, Kopczynski CC (2015). Effect of AR-13324 on episcleral venous pressure in Dutch belted rabbits. J. Ocul. Pharmacol. Ther..

[14] Reitsamer HA, Kiel JW (2002). A rabbit model to study orbital venous pressure, intraocular pressure, and ocular hemodynamics simultaneously. Invest. Ophthalmol. Vis. Sci..

[15] Verkicharla PK, Ohno-Matsui K, Saw SM (2015). Current and predicted demographics of high myopia and an update of its associated pathological changes. Ophthalmic Physiol. Opt..

[16] Chen Z (2018). Schlemm’s canal and trabecular meshwork morphology in high myopia. Ophthalmic Physiol. Opt..

[17] Carreon T, van der Merwe E, Fellman RL, Johnstone M, Bhattacharya SK (2017). Aqueous outflow: A continuum from trabecular meshwork to episcleral veins. Prog. Retin. Eye Res..

[18] Hayashi M, Ito Y, Takahashi A, Kawano K, Terasaki H (2013). Scleral thickness in highly myopic eyes measured by enhanced depth imaging optical coherence tomography. Eye (Lond).

[19] Chen W, Wang Z, Zhou X, Li B, Zhang H (2012). Choroidal and photoreceptor layer thickness in myopic population. Eur. J. Ophthalmol..

[20] Verkicharla PK, Mathur A, Mallen EA, Pope JM, Atchison DA (2012). Eye shape and retinal shape, and their relation to peripheral refraction. Ophthalmic Physiol. Opt..

[21] Ohno-Matsui K, Jonas JB (2019). Posterior staphyloma in pathologic myopia. Prog. Retin. Eye Res..

[22] Ohno-Matsui K (2021). IMI pathologic myopia. Invest. Ophthalmol. Vis. Sci..

[23] Ohno-Matsui K, Ikuno Y, Lai TYY, Gemmy Cheung CM (2018). Diagnosis and treatment guideline for myopic choroidal neovascularization due to pathologic myopia. Prog. Retin. Eye Res..

[24] Ohno-Matsui K (2015). International photographic classification and grading system for myopic maculopathy. Am. J. Ophthalmol..

[25] Tanihara H (2016). One-year clinical evaluation of 0.4% ripasudil (K-115) in patients with open-angle glaucoma and ocular hypertension. Acta Ophthalmol..

[26] Sato S, Hirooka K, Nitta E, Ukegawa K, Tsujikawa A (2016). Additive intraocular pressure lowering effects of the rho kinase inhibitor, ripasudil in glaucoma patients not able to obtain adequate control after other maximal tolerated medical therapy. Adv. Ther..

[27] Inoue K, Ishida K, Tomita G (2018). Effectiveness and safety of switching from prostaglandin analog monotherapy to prostaglandin/timolol fixed combination therapy or adding ripasudil. Jpn. J. Ophthalmol..

[28] Tanihara H, Kakuda T, Sano T, Kanno T, Kurihara Y (2022). Long-term intraocular pressure-lowering effects and adverse events of ripasudil in patients with glaucoma or ocular hypertension over 24 months. Adv. Ther..

[29] Tanihara H, Kakuda T, Sano T, Kanno T, Gunji R (2020). Safety and efficacy of ripasudil in Japanese patients with glaucoma or ocular hypertension: 12-month interim analysis of ROCK-J, a post-marketing surveillance study. BMC Ophthalmol..

[30] Sakata R (2021). The additive effect of ROCK inhibitor on prostaglandin-treated japanese patients with glaucoma indicating 15 mmHg and under: ROCK U-15. Adv. Ther..

[31] Maruyama Y (2020). Safety and efficacy of long-term ripasudil 0.4% instillation for the reduction of intraocular pressure in japanese open-angle glaucoma patients. J. Ocul. Pharmacol. Ther..

[32] Inoue K, Okayama R, Shiokawa M, Ishida K, Tomita G (2018). Efficacy and safety of adding ripasudil to existing treatment regimens for reducing intraocular pressure. Int. Ophthalmol..

[33] Inazaki H (2017). Efficacy of the additional use of ripasudil, a rho-kinase inhibitor, in patients with glaucoma inadequately controlled under maximum medical therapy. J. Glaucoma.

[34] Inazaki H (2017). One-year efficacy of adjunctive use of Ripasudil, a rho-kinase inhibitor, in patients with glaucoma inadequately controlled with maximum medical therapy. Graefes Arch. Clin. Exp. Ophthalmol..

[35] Matsumura R, Inoue T, Matsumura A, Tanihara H (2017). Efficacy of ripasudil as a second-line medication in addition to a prostaglandin analog in patients with exfoliation glaucoma: A pilot study. Clin. Drug Investig..

[36] Wang YX (2023). High myopia as risk factor for the 10-year incidence of open-angle glaucoma in the Beijing Eye Study. Br. J. Ophthalmol..

[37] Ohno-Matsui K (2011). Long-term development of significant visual field defects in highly myopic eyes. Am. J. Ophthalmol..

[38] Holden BA (2016). Global prevalence of myopia and high myopia and temporal trends from 2000 through 2050. Ophthalmology.

[39] Fang Y (2021). Novel paravascular lesions with abnormal autofluorescence in pathologic myopia. Ophthalmology.

[40] Xiong J (2022). Papillary and peripapillary hemorrhages in eyes with pathologic myopia. Invest. Ophthalmol. Vis. Sci..

[41] Ohno-Matsui K, Lai TY, Lai CC, Cheung CM (2016). Updates of pathologic myopia. Prog. Retin. Eye Res..

[42] Yoshida T (2023). Outcomes of standalone ab interno trabeculotomy in the treatment of open-angle glaucoma in eyes with high myopia. BMC Ophthalmol..

[43] Hahn SR, Kotak S, Tan J, Kim E (2010). Physicians’ treatment decisions, patient persistence, and interruptions in the continuous use of prostaglandin therapy in glaucoma. Curr. Med. Res. Opin..

